# Tendon-inspired anti-freezing tough gels

**DOI:** 10.1016/j.isci.2021.102989

**Published:** 2021-08-17

**Authors:** Sidi Duan, Shuwang Wu, Mutian Hua, Dong Wu, Yichen Yan, Xinyuan Zhu, Ximin He

**Affiliations:** 1 Department of Materials Science and Engineering, University of California Los Angeles, Los Angeles, CA 90095, USA; 2 School of Chemistry and Chemical Engineering, State Key Laboratory of Metal Matrix Composites, Shanghai Jiao Tong University, Shanghai 200240, China

**Keywords:** biomimetics, materials property, polymers

## Abstract

Hydrogels have gained tremendous attention due to their versatility in soft electronics, actuators, biomedical sensors, etc. Due to the high water content, hydrogels are usually soft, weak, and freeze below 0°C, which brings severe limitations to applications such as soft robotics and flexible electronics in harsh environments. Most existing anti-freezing gels suffer from poor mechanical properties and urgently need further improvements. Here, we took inspirations from tendon and coniferous trees and provided an effective method to strengthen polyvinyl alcohol (PVA) hydrogel while making it freeze resistant. The salting-out effect was utilized to create a hierarchically structured polymer network, which induced superior mechanical properties (Young's modulus: 10.1 MPa, tensile strength: 13.5 MPa, and toughness: 127.9 MJ/m^3^). Meanwhile, the cononsolvency effect was employed to preserve the structure and suppress the freezing point to −60°C. Moreover, we have demonstrated the broad applicability of our material by fabricating PVA hydrogel-based hydraulic actuators and ionic conductors.

## Highlights

• PVA gels with extreme low temperature tolerance and superior mechanical properties
• Fabrication incorporates ice templating, salting-out, and cononsolvency effect
• Anisotropic hierarchical structure resembles tendon at multiple length scales
• Anti-freezing tough gel demonstrated as hydraulic actuators and ionic conductors

## Graphical abstract

**Figure:**
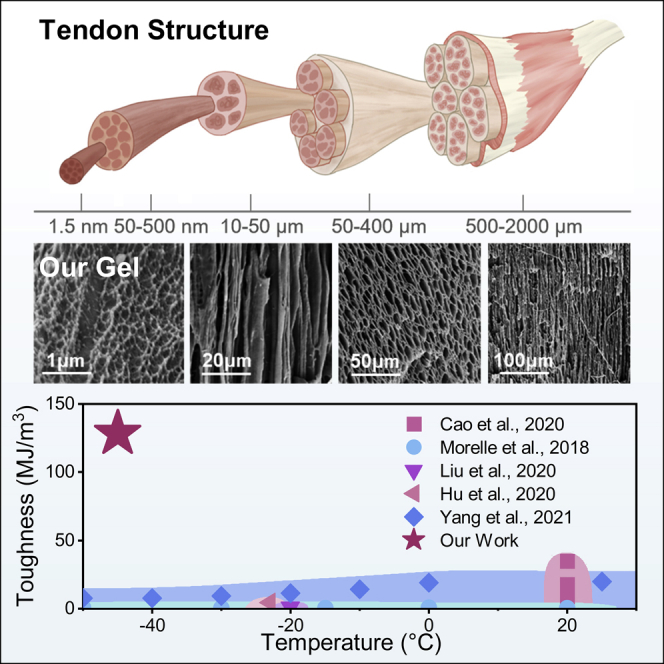

## Introduction

Hydrogels are three-dimensional (3D) networks of hydrophilic polymers holding a large amount of water (Ahmed, 2015). Due to the superior softness, responsiveness, and biocompatibility, hydrogels find applications in drug delivery (Li and Mooney, 2016), tissue engineering (Billiet et al., 2012), soft electronics (Yang and Suo, 2018), biomedical sensors (Tavakoli and Tang, 2017), actuators (Ionov, 2014), soft robotics (Liu et al., 2020a), etc. However, because of the low polymer density and high water content, hydrogels fail to meet mechanical property requirements in many important fields such as artificial tissue, actuator, and soft robotics. In order to toughen the typically soft and weak hydrogels, researchers developed various methods such as introducing anisotropic structures (Zhang et al., 2014), compositing (Chen et al., 2020), double network (Sun et al., 2012), mechanical training (Lin et al., 2019), thermal annealing (Owusu-Nkwantabisah et al., 2018), and ice templating (Zhang et al., 2005). However, the mechanical properties of materials fabricated via the methods mentioned above are far from satisfactory. Meanwhile, many soft natural materials found in animal bodies sometimes possess superior properties and bring insights into artificial material development. Tendon, for example, is a cord of tough and fibrous tissue that attaches muscle to bone. The major components of tendons are collagens, combining to form microfibrils, which then bundle into fibrils. Fibrils group together to become fibers, which then constitute fiber bundles and ultimately come together to form fascicles (Benjamin et al., 2008). This anisotropic hierarchical structure endows tendon with superior mechanical properties. Inspired by the sophisticated structures of tendon, tough hydrogels have been successfully fabricated through the synergistic effect of ice templating and salting out (Hua et al., 2021).

Apart from low mechanical properties, the high content of water in the hydrogel brings another issue. Water will freeze at subzero temperatures, causing hydrogel to lose its stretchability and nullifying functionalities such as actuation, stimuli sensing, and conductivity. The wisdom of nature can also direct us to design anti-freezing gels. Some homothermic animals, such as polar bears, can survive in extremely cold weather thanks to their thick fur and body heat generation (Cui et al., 2018). Poikilotherms and plants living in cold habitats can also stay alive under subzero temperatures. They secret substances to suppress the ice nucleation and growth instead of producing heat through metabolism (Worland et al., 1992). For instance, coniferous trees can survive in −40°C environments for long periods owing to some cryoprotective metabolites (sugars, starch, dehydrin proteins, lipids, etc.) to lower the freezing point (Xu et al., 2020). Inspired by the poikilotherms and plants, cosolvents (Wu et al., 2021), ionic liquids (Wang et al., 2020), and salts (Morelle et al., 2018) have been utilized to depress the freezing point. However, these freezing point depression methods are not very effective in enhancing mechanical properties, and some methods even weaken the hydrogels (Sadeghi and Jahani, 2012).

Herein, we proposed a method of fabricating tough polyvinyl alcohol (PVA) hydrogel with a low freezing point by incorporating both salting-out and cononsolvency effects. Directional freezing of PVA precursor generates aligned pores in the polymer matrix, while the introduction of salt ions induces the strong aggregation of polymer chains and further generates nano-structures and micro-structures. The resultant hierarchical structure at multiple length scales endows the PVA gel with superior mechanical properties. Unfortunately, salts that exhibit a strong salting-out effect on polymer chains usually have weak freezing point depression ability in aqueous solutions (Hitchcock and Dougan, 1935; Sadeghi and Jahani, 2012), and the salting-in salts can depress the freezing point but compromise the mechanical properties. To maintain the superior mechanical performance of the gels, the cononsolvency effect is utilized. The solubility of a macromolecule can decrease in the mixture of two good solvents, and this promotes polymer chain aggregation and crystalline domain formation (Scherzinger et al., 2014). Dimethyl sulfoxide (DMSO) and water mixture have cononsolvency effect on PVA (Wu et al., 2021; Alsaid et al., 2021). While maintaining the hydrogen bonding and the structures generated by salting out, using mixed solvents can effectively lower the freezing point to −60°C at 60 wt% DMSO mixing ratio (eutectic point, phase diagram in Figure S1), enabling the anti-freezing property of the presented gel (Sato and Hukuda, 1962).

At room temperature, this material exhibited Young's modulus of 7.6 MPa, the tensile strength of 13.3 MPa, and toughness of 110.5 MJ/m^3^, making it 5.5 times tougher than the toughest anti-freezing gel (Yang et al., 2021) and over 10 times tougher than tendon (Maganaris and Narici, 2005). The exchange of liquid in gel from salt solution to DMSO/H_2_O mixture endowed the gel with superior mechanical performances at extremely low temperatures. This PVA gel could remain flexible at −60°C, and its mechanical properties measured at −45°C are even slightly higher than at room temperature (Young's modulus: 10.1 MPa, tensile strength: 13.5 MPa, and toughness: 127.9 MJ/m^3^). Apart from being tough, this hydrogel is also tear resistant, enabled by the aligned polymer fibers bridging the cracks. A hydraulic actuator was then made from this material by molding, which demonstrated high loading weight (100 g) at both room temperature and −45°C. Furthermore, we showed that this material exhibited anisotropic ionic conductivity after adding sulfuric acid into the liquid. This anti-freezing tough gel opens up new possibilities for the application of hydrogels as actuators, soft robotics, and flexible electronics under subzero temperatures.

## Results and discussion

### Fabrication and structure

**Figure 1:**
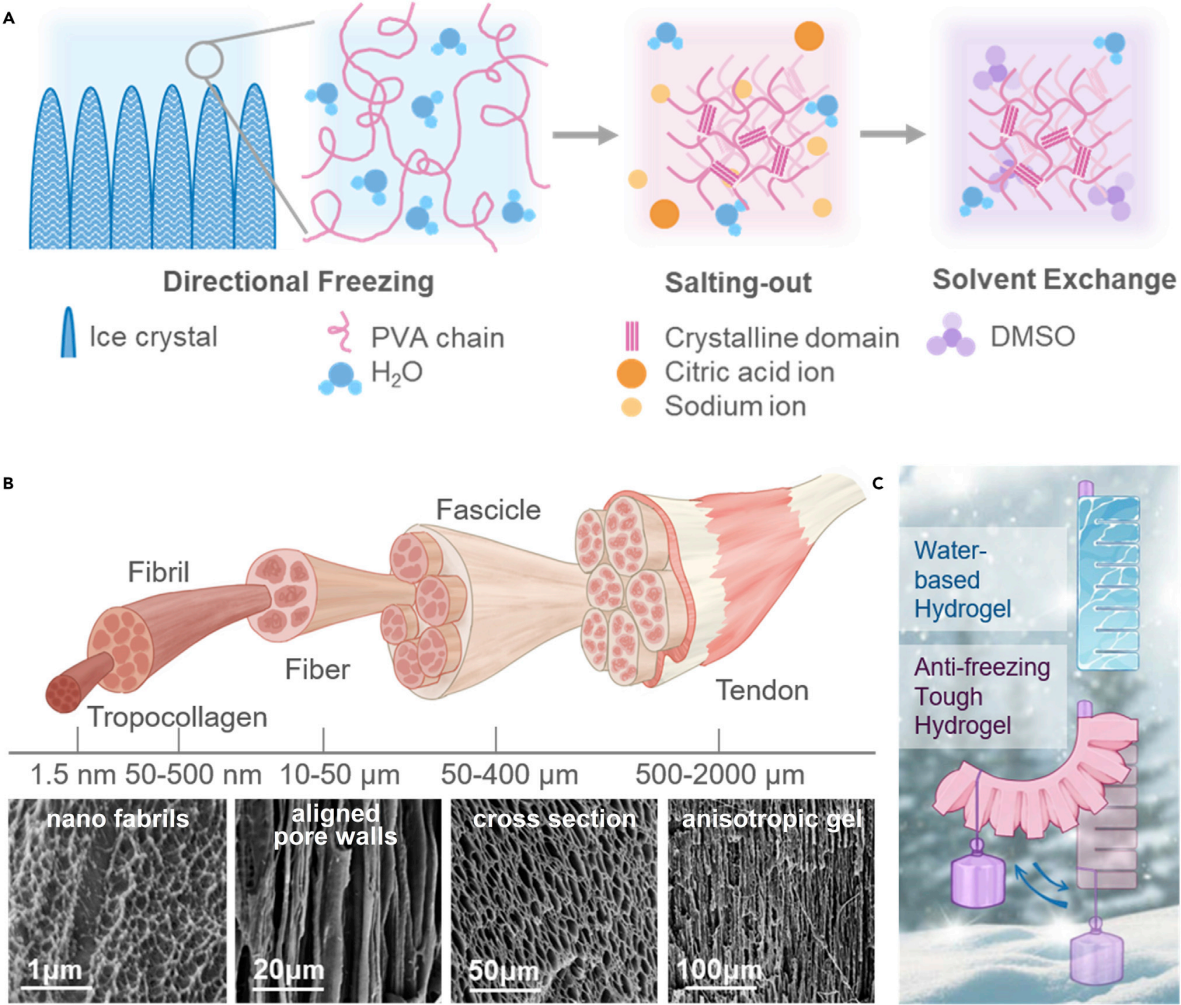
The schematic of fabrication steps and hierarchical structures of tendon and anti-freezing tough gel (A) Schematic of the fabrication steps of the tendon-inspired anti-freezing tough gel. (B) Illustration of tendon structures and SEM images of the anti-freezing tough gel. (C) Illustration indicating the high mechanical performance and freeze resistance of our material.

Tendons are strong cords of fibrous material that transmit force from muscles to bones, and they are capable of withstanding high stress in the loading direction. The hierarchical and anisotropic structures contribute to the remarkable mechanical properties. Inspired by such complex anisotropic structures, we fabricated the comparable complex structures in the anti-freezing PVA gel by utilizing the salting-out effect and cononsolvency effect. As shown in Figure 1A, the PVA precursor first went through directional freezing, during which the ice crystals expelled the polymer chains to areas between ice columns. In this way, these PVA chains were condensed into a through-height honeycomb structure. The scanning electron microscopy (SEM) images in Figure 1B show the aligned pore walls and cross-sectional view of this structure. Next, the frozen gel was placed into a 1.5 M sodium citrate (SC) solution, which has a strong salting-out effect where the high ionic strength of salt ions reduces the solubility of polymers (Baldwin, 1996). The introduction of ions induced more hydrogen bonds between PVA chains, which increased the degree of crystallinity and further aggregated the PVA chains. The PVA hydrogel was then soaked into a 60 wt% DMSO/40 wt% H_2_O (DMSO/H_2_O, DH) mixture. This mixture of solvents well maintained the mechanical properties and made the gels freeze tolerant by lowering the freezing point. The resultant anisotropic anti-freezing tough gel is denoted as aSC-DH gel. The final structure of our PVA gel resembled the aligned hierarchical structure of tendon. Figure 1B depicts the structures of tendon on multiple length scales and also shows the SEM images of our PVA gel under different magnification. Tendon has nanometer-scale collagen strands and fibrils and micron-scale fibers and fascicles. Similarly, our PVA gel has nano-fibrils and aligned walls with pore diameter of around 10 microns. The resemblance between this artificial hydrogel and natural tendon material indicates the versatility of hydrogel materials.

### Mechanical properties

**Figure 2:**
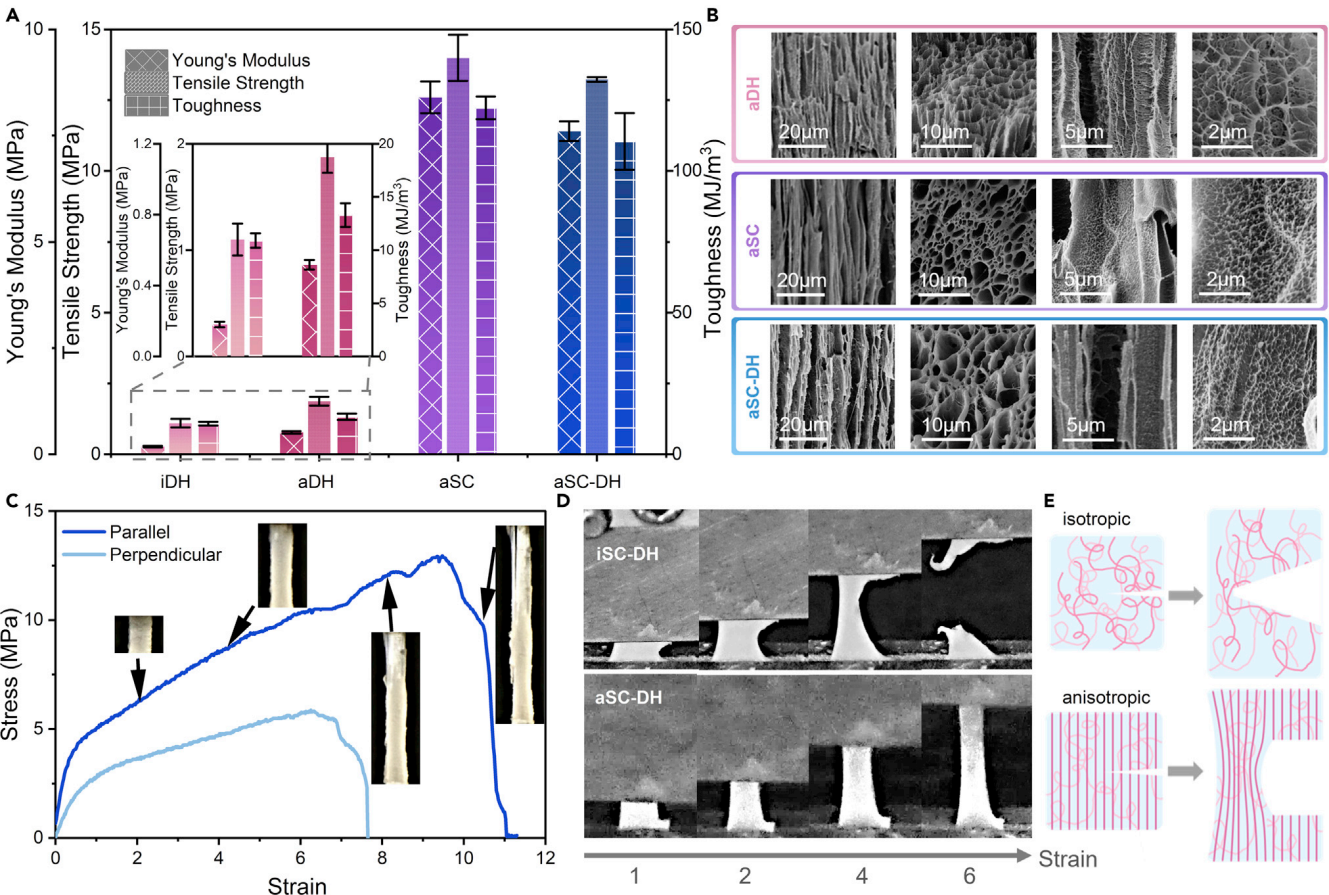
Mechanical properties and morphologies of PVA gels went through different fabrication steps (A) Comparison of Young's modulus, tensile strength, and toughness of isotropic DMSO/H_2_O (iDH), anisotropic DMSO/H_2_O (aDH), anisotropic sodium citrate (aSC), and anisotropic sodium citrate > DMSO/H_2_O (aSC-DH) gels. (B) SEM images of (A) aDH, (B) aSC, and (C) aSC-DH gels. (C) Stress-strain curves for aSC-DH gels in parallel and perpendicular directions. The insets are photos showing the samples with different strains. (D) Photos taken during the notched tensile tests of isotropic SC-DH gel and anisotropic SC-DH gel in parallel direction. (E) Illustrations of crack propagation in isotropic and anisotropic samples.

Figure 2A shows Young's modulus, tensile strength, and toughness of four different gels prepared differently, including the isotropic DMSO/H_2_O (iDH) gel (gelation by the solvent mixture following homogeneous freezing), the anisotropic DMSO/H_2_O (aDH) gel (mixture of solvents following directional freezing), the anisotropic sodium citrate (aSC) gel (salting out following directional freezing), and the anisotropic sodium citrate > DMSO/H_2_O (aSC-DH) gel (mixture of solvents following salting out after directional freezing). If not stated otherwise, all PVA precursors contain 10 wt% PVA powder of Mw. 89,000-98,000 and 99% degree of hydrolysis (see method details section for reason). The iDH gel was fabricated by simple mixing of PVA solutions in DMSO and H_2_O, as described in (Wu et al., 2021). The aDH gel was obtained by directionally freezing the PVA precursor and then soaking the frozen gel in DMSO/H_2_O mixture. The comparison between iDH and aDH gels indicates the effectiveness of the directional freezing process in enhancing mechanical properties. The Young's modulus had a 3-time increase (from 0.18 MPa to 0.52 MPa), tensile strength was nearly doubled (from 1.1 MPa to 1.9 MPa), and toughness was slightly increased from 10.9 MJ/m^3^ to 13.3 MJ/m^3^ (Figure 2A). Meanwhile, the salting-out effect of sodium citrate solution also greatly improved the mechanical performance. The aSC gels, which went through the salting-out process in sodium citrate solution, had Young's modulus, tensile strength, and toughness of 8.4 MPa, 14.0 MPa, and 122.3 MJ/m^3^ (Figure 2A), respectively. These values increased 10 times over the aDH gels without salting-out treatment, which showed that sodium citrate solution has a strong capability of strengthening PVA hydrogels. Subsequently, these aSC gels, after soaking in water for two days, suffered a drastic drop in mechanical properties. Specifically, Young's modulus, ultimate tensile strength, and toughness decreased by 82%, 63%, and 78%, respectively (Figure S2). By contrast, if soaking the aSC gels in DMSO/H_2_O mixed solvents instead of pure water as above, the resulting aSC-DH gels had only a 10% drop in properties after the solvent exchange (Figure 2A). This indicates that the exchange of liquid in the gel to DMSO/H_2_O did not pose much effect on the structures (Figure 2B). Specifically, Young's modulus dropped from 8.4 MPa to 7.6 MPa, tensile strength changed from 14.0 MPa to 13.2 MPa, while toughness decreased from 122.3 MJ/m^3^ to 110.5 MJ/m^3^. This property contrast between the results of soaking with water vs. DMSO/H_2_O mixed solvents indicates the effectiveness of cononsolvency effect in preserving the mechanical properties of salting-out-toughened PVA gels.

### Morphologies

The morphology studies shown in Figure 2B corroborate the mechanical testing results. When the PVA aqueous solution first went through directional freezing, PVA chains were condensed into a through-height honeycomb structure. When the frozen precursor was directly soaked in the DMSO/H_2_O mixture, the cononsolvency effect took charge in aggregating polymer chains in the regions between ice crystals. The resulting aDH gel had nanometer-sized holes on the pore walls (Figure 2B). When the frozen gel was placed into sodium citrate solution instead of DMSO/H_2_O, these ions caused the polymer walls to become more condensed with no visible hole on the walls and created nanofibril patterns on the walls of aSC gels. It indicates that the cononsolvency effect was weaker than the salting-out effect in aggregating PVA. The PVA hydrogel was then soaked into DMSO/H_2_O mixture for 7 days for a complete solvent exchange. After that, the hierarchical structure across multiple length scales was still well preserved (Figure 2B). Although DMSO/H_2_O mixture could not induce an as dense structure or nanofibrils as the sodium citrate solution could, this solvent mixture would not disassemble the tight polymer aggregates and dense polymer walls formed through the salting-out process. These results agree with the solid content measurement, where the aDH gel has the lowest solid content of 12%, the aSC gel has the highest solid content of 34%, and the solid content of aSC-DH gel is 28% (Figure S12). Combining the results from the mechanical tests and the SEM images, we concluded that the superior mechanical performance was attributed to both the aligned structures and strong aggregation between polymer chains, which resulted from directional freezing and salting out, respectively. Meanwhile, cononsolvency effect could largely maintain the mechanical properties during the solvent exchange. Hierarchically, the salt ions greatly strengthened the material at molecular and nanometer scale, while the ice crystals created an aligned structure that strengthened the material at the micrometer level.

### Anisotropic properties

The stress-strain curves of tensile tests for aSC-DH gels in the directions parallel and perpendicular to the freezing direction are shown in Figure 2C. The gradual fracture mode and fiber pull out are both commonly observed behaviors in anisotropic polymer systems (Zuo et al., 2021). The alignment not only enhanced strength in the parallel direction but also ensured the resistance to crack propagation in the polymer matrix. Figure 2D shows the distinct fracturing processes of two different gels during the tensile tests. The isotropic PVA gel (iSC-DH-freeze-thaw) sample was made by the homogeneous freeze-thaw method, with a horizontal cut in the middle from the right side. When this sample was elongated, this initial crack readily propagated through the left half of the gel, and the sample failed at around 500% strain. By contrast, the aSC-DH gel with aligned structures did not exhibit any sign of crack propagation, instead, the right half with the initial crack separated, while the left unnotched half elongated as if there was no crack. This indicates that the condensed aligned fibers can pin and bridge the crack due to their high strength, and the subsequent stretching will cause fiber pull out (Figure 2C). Crack propagation will begin when the fibers start to fracture, which requires much higher stress than isotropic gels since the fibers are denser and better aligned (Theocaris and Stassinakis, 1981).

### Anti-freezing performance

**Figure 3:**
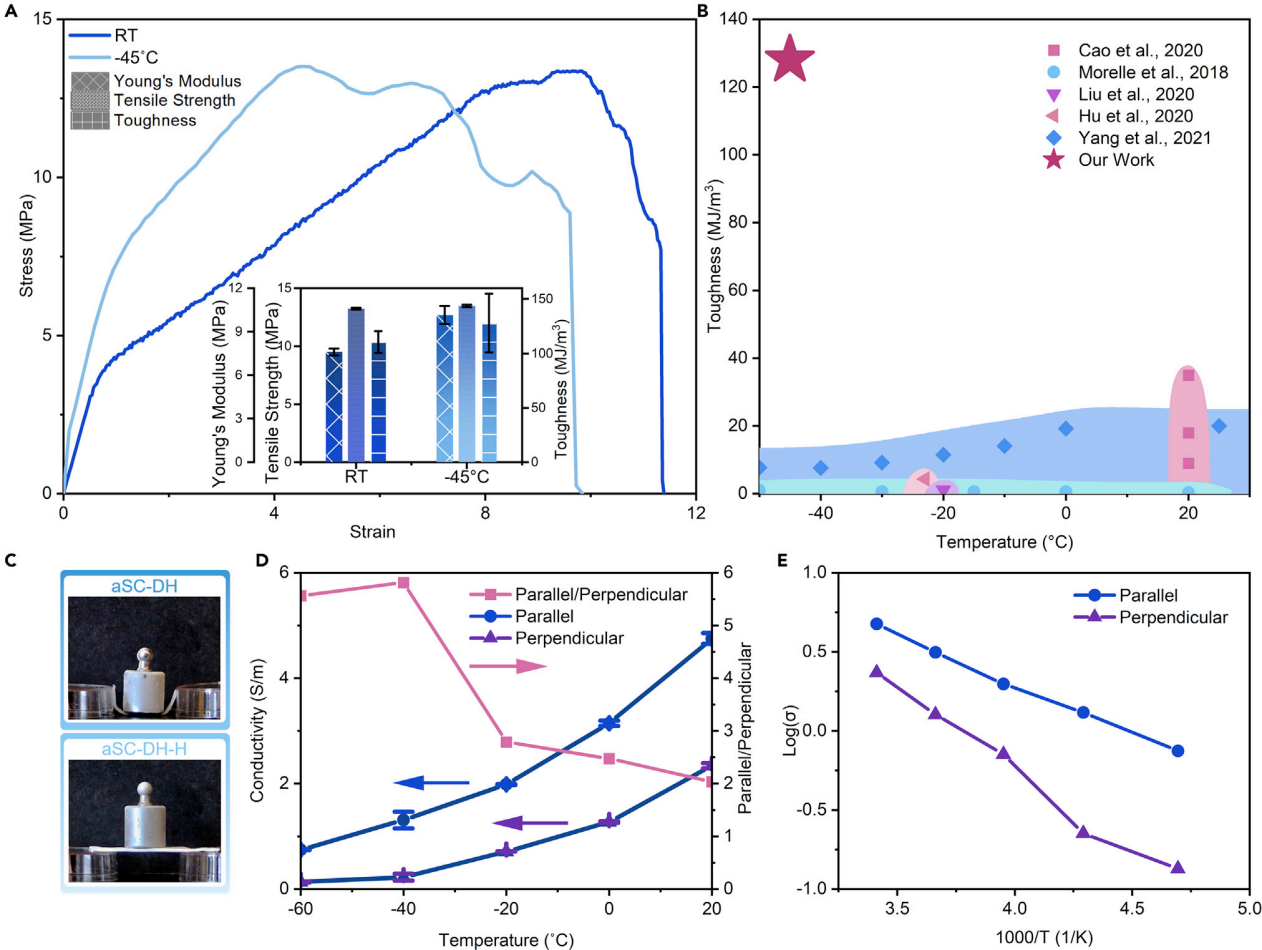
Low-temperature performances of aSC-DH hydrogel (A) Stress-strain curves for tensile tests of aSC-DH hydrogel at room temperature and −45°C. Inset is the comparison of Young's modulus, tensile strength, and toughness between aSC-DH gels at room temperature and −45°C. (B) Comparison of lowest operation temperature and toughness under these temperatures with other works. (C) (Top) Photo showing aSC-DH hydrogel remained flexible under −60°C. (Bottom) Photo showing aSC-DH-H gel became rigid under −60°C. (D) A plot of conductivities of aSC-DS gels in parallel and perpendicular directions under various temperatures, and plot of the ratio of parallel conductivity over perpendicular conductivity. (E) Arrhenius plot of conductivities of aSC-DS gels in parallel and perpendicular directions under various temperatures.

The anti-freezing performance of aSC-DH gel was examined by several tests at subzero temperatures. The tensile test was conducted in a −45°C cooling chamber, and the result was compared with the results at room temperature (RT) (Figure 3A). The cooled samples exhibited higher Young's modulus (10.1 MPa), similar tensile strength (13.4 MPa), and slightly higher toughness (127.9 MJ/m^3^) compared to samples at RT (7.6 MPa, 13.2 MPa, and 110.5 MJ/m^3^). Hydrogel matrixes contain a large amount of covalent and hydrogen bonds, and during deformation, polymer chains are straightened while hydrogen bonds go through dissociation and reassociation (Hu et al., 2017). At low temperatures, polymer chains are harder to slide past each other, and this results in higher Young's modulus (Cebon and Cheung, 2015). Moreover, the freeze resistance of DMSO/H_2_O ensured large fracture strain. Both the high modulus and large strain contributed to the exceptional toughness at low temperatures. The photos in Figure 3C more intuitively show the anti-freezing capability of our material. The aSC-DH-H gel went through the same fabrication steps as the anti-freezing aSC-DH gel, but it was soaked in water at last. Shortly after this sample was taken out of a −60°C bath, a 50 g weight cooled to the same temperature was placed on top. This photo demonstrates the rigidity of the frozen gel, and it indicates that any conventional water-based hydrogel would be rendered useless under such conditions. On the contrary, aSC-DH gel retained its flexibility after being cooled to −60°C. Compared to other works on freeze-resistant tough hydrogels shown in Figure 3B, our material has superior mechanical performances at low temperature (Cao et al., 2020; Morelle et al., 2018; Liu et al., 2020b; Hu et al., 2020; Yang et al., 2021). This aSC-DH gel has over 10-time higher toughness than other works at low temperature (−45°C).

Moreover, high conductivity at low temperatures was realized by incorporating 2 M sulfuric acid in the DMSO/H_2_O mixture. Like the anisotropic mechanical properties, the conductivities of this aSC-DS gel in the directions parallel and perpendicular to the pore alignment, respectively, are also different. Figure 3D shows the conductivities in these two directions under various temperatures. In parallel direction, the aligned pores served as channels for ions to transport with low resistance (Lin et al., 2019), while in the perpendicular direction, ions had to pass through dense pore walls. Such a structural anisotropy led to conductivity anisotropy. At different temperatures, the difference ranged from 2 to 6 times. Variation of ionic conductivity of liquids with the temperature usually follows Arrhenius relation (Vila et al., 2007):

(Equation 1) $$\sigma =\sigma_{0}e^{-\frac{E}{kT}}$$

where σ is conductivity, $\sigma_{0}$ is a prefactor, E is the activation energy for the whole process, k is Boltzmann constant, and T is temperature. The Arrhenius plot indicates that the activation energy terms in Equation 1 for the two directions are different and will lead to a greater difference in conductivity at lower temperatures. Apart from the difference in conductivity in the two directions, the anisotropic structure can also enhance the conductivity of PVA gel compared to the isotropic PVA gel. To demonstrate the higher conductivity, we built a circuit with LED lights and connected the anisotropic or isotropic gels with the same sizes in the circuit as shown in Figure S8. The experiment was conducted at room temperature and −20°C. A cyclic voltage scan from -5V to 5V was applied on the circuit, and the brightness of light with anisotropic gel and isotropic gel as conductors was compared. As shown in Figure S9, the LED lights with anisotropic gels as conductors at both room temperature and −20°C are brighter than the LED lights with isotropic gels as conductors. The higher electrical conductivity of anisotropic gel can be confirmed by the C-V curve, which clearly shows the higher current for anisotropic gels than the isotropic ones under the same voltage. The addition of sulfuric acid will not affect the mechanical properties of the conductive gel, as plotted in Figure S10. The exceptional electrical properties bring potential utilization of this material in, for instance, subzero temperature electronics and sensors capable of withstanding harsh environments both mechanically and temperature wise.

### Hydraulic actuator made by anti-freezing tough gel

**Figure 4:**
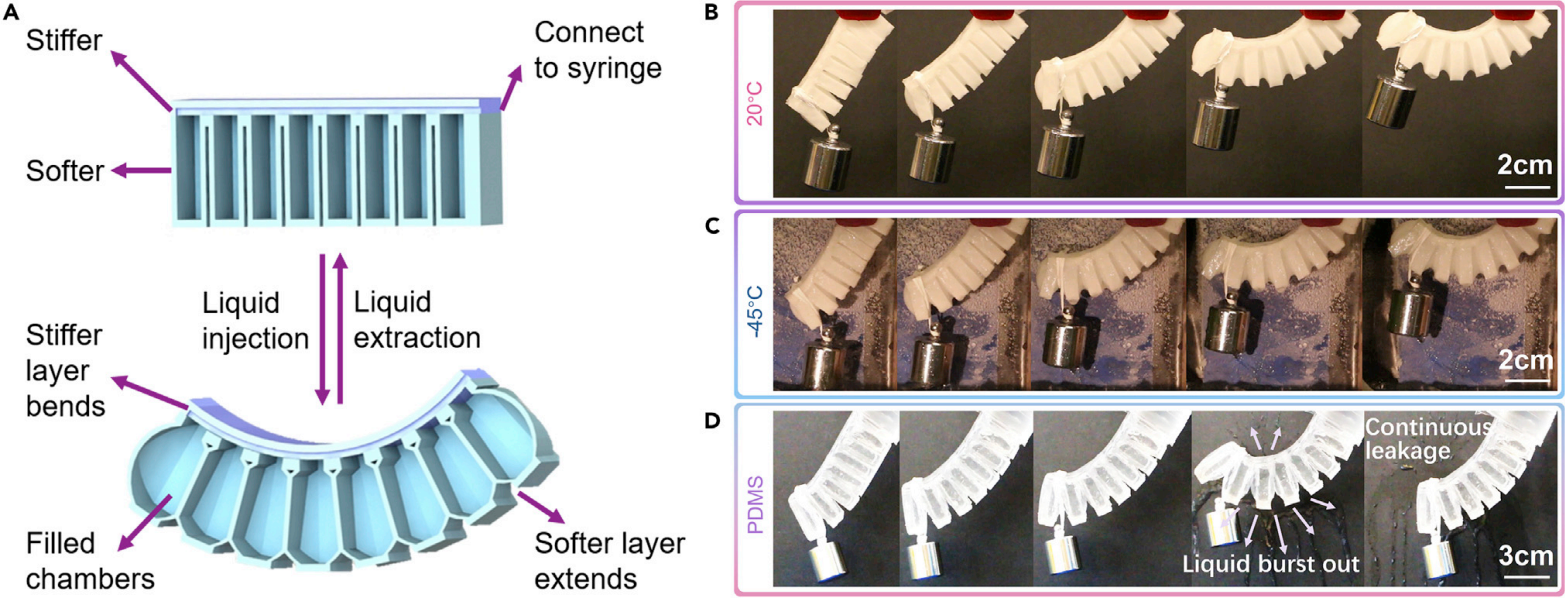
PVA hydraulic actuator (A) Schematic cross-sectional view of the hydraulic actuator at unpressurized and pressurized states. (B) Photos of the PVA hydraulic actuator lifting a 100 g weight in air upon liquid injection at 20°C. (C) Photos of the PVA hydraulic actuator lifting a 100 g weight in air upon liquid injection at −45°C. (D) Photos of a PDMS actuator lifting a 100 g weight in air upon liquid injection at 20°C. The PDMS actuator broke at the fourth frame and was no longer active in the fifth frame.

Compared to stimuli-responsive actuators and actuators incorporating active elements, hydraulic actuators' advantages lie in fast response rate and high actuation force (Liu et al., 2020a). In fact, the actuating force has a linear relationship with applied pressure, which means that the strength of material directly determines the maximum force the actuator can provide (Yuk et al., 2017). Based on the presented high load-bearing ability, tear resistance, and low freezing point, our material was made into hydraulic actuators that can lift heavy weight at both room temperature and subzero temperatures. In order for the actuator to bend effectively, the material on the back of the actuator should have higher stiffness than the other parts (Figure 4A) because the stiffer back strip will then bend without extending while the softer chamber walls bulge. To achieve this difference in stiffness, a 10 wt% PVA precursor was used to build the softer part of this actuator, and a 15 wt% PVA precursor was used for the stiffer part. This actuator structure was fabricated by freeze-thaw molding (Figure S3). First, the 10 wt% PVA went through 5 freeze-thaw cycles; then, the 15 wt% PVA was poured on top of the bottom softer part before going through another 2 freeze-thaw cycles. The seamless and strong adhesion between the two parts was realized by molecular topology adhesion, where the PVA chains penetrated into the bottom part, formed physical entanglement, and stitched the two parts together (Steck et al., 2020). The actuator can bend upwards upon liquid injection in air and liquid, with a 100g weight tied to the end of the actuator (Figures 4B and S4A; Videos S1, S2, S3, and S4). To demonstrate low-temperature performance both in air and in solution, the actuator was then placed in a −45°C cooling chamber and a −60°C cooling bath, respectively. Results show that with the same amount of DMSO/H_2_O solution injected, the actuator could lift the weight to the same height as at room temperature (Figures 4C and S4B); in other words, the material was as robust at low temperatures as at room temperature. For comparison, an actuator of a similar design was fabricated with polydimethylsiloxane (PDMS), a commonly used elastomer for most reported hydraulic actuators. In weight-lifting tests, upon injection of liquid, the PDMS actuator bent but broke and leaked before reaching the same degree of deformation as the PVA actuator did (Video S5). The photo in Figure 4D captures this process when the PDMS actuator fractured because of large deformation and pressure, and the liquid inside burst out. This PDMS actuator could no longer lift the weight after the leakage. Furthermore, to quantitatively demonstrate the actuation ability, we tested the actuation force, which reached over 20 N before failure (Figure S6), 5 times higher (normalization by size) compared to state-of-the-art hydrogel hydraulic actuator (Yuk et al., 2017). These results proved that PVA hydrogels could be strengthened to produce high actuation force without failure, even outperforming commonly used commercial elastomers. Moreover, the actuator can be further strengthened in specific directions by directionally freezing the PVA precursor before salt solution treatment instead of freeze-thawing the precursor. As shown in Figure S7, since the weakest direction in the anisotropic gel (aSC-DH-perpendicular) is still stronger than the isotropic material fabricated for the actuator (iSC-DH-freeze-thaw), the pressure enduring ability of the actuator will not be compromised after this anisotropic strengthening. Based on specific application situations, the anisotropic structure can be introduced to enhance load-bearing and tear-resistant abilities in the desired direction. Apart from PVA, it is anticipated that other types of hydrogels made of broad choices of polymers, including biopolymers alginate and gelatin, may be created and toughened by this presented method, as demonstrated in our recent report (Hua et al., 2021).

Video S1. PVA actuator actuates in air at 20°C, related to Figure 4

Video S2. PVA actuator actuates in air at −45°C, related to Figure 4

Video S3. PVA actuator actuates in DMSO/H2O at 20°C, related to Figure 4

Video S4. PVA actuator actuates in DMSO/H2O at −60°C, related to Figure 4

Video S5. PDMS actuator actuates in air at 20°C, related to Figure 4

### Conclusions

In summary, we provide an effective way of achieving high toughness and freeze resistance at the same time in PVA gel. Existing hydrogel materials either are strengthened but become rigid at temperatures well below 0°C or remain flexible at sub-zero temperatures but have poor mechanical properties. In order to target these two challenges in the same material, we took inspiration from natural materials, namely tendon and coniferous trees, to enhance mechanical properties and depress the freezing point of PVA gel. The tendon-mimicking anisotropy structure created by the combination of directional freezing and salting-out effect strengthens the polymer, as well as endows PVA gel with tear resistance. Changing the liquid in the gel to low-freezing-point DMSO/H_2_O simultaneously maintained the mechanical properties and endowed the gel with anti-freezing capability. Such gels exhibited high Young's modulus, tensile strength, and toughness under a broad range of temperatures down to −45°C. The operating temperature is among the lowest of all anti-freezing gels, and the mechanical properties are well beyond other anti-freezing gels. Moreover, we demonstrated that our material is competent for low-temperature actuation and weight lifting, and the performance surpassed that of the PDMS actuator. Apart from being mechanically outstanding, our material can also become anisotropic ionic conductors over a wide temperature range. These exceptional properties and performances open up possibilities of utilizing this material for soft robotics and soft electronics that operate in cold environments.

### Limitations of the study

Two issues are inevitable for hydrogel materials: liquid permeation and liquid evaporation. Since hydrogels swell in their solvents, liquid molecules can diffuse at specific rates in the polymer matrix. When the concentration or pressure on two sides of a piece of hydrogel is different, there would be a net flow of liquid from one side to the other side. However, (Yuk et al., 2017) have proven that this leakage rate is several orders of magnitude slower than the liquid supply flow rate during actuation. Thus, we can safely say that the liquid permeation rate is negligible. Oftentimes, the hydrogels find application in the atmospheric environment. This is not much of a concern for hydraulic actuators in use because there will be a constant liquid supply to the hydrogel. Due to the low vapor pressure of DMSO and water mixture (Catalán et al., 2001), the gel will remain hydrated for short-term use in other applications. If long-term exposure or contact with other solvents is expected, the gels should require encapsulation. The encapsulating material needs to be highly stretchable since the strain before fracture of our material is more than 1000%. Ecoflex, for example, is an option for encapsulating material.

Although DMSO is a widely used cryoprotectant in cryopreservation (Awan et al., 2020), the toxicity of DMSO has always been an issue for debate. DMSO can impose negative effects on cells at high dosage and long-term use, which brings concerns about the toxicity of DMSO in certain applications. An alternative mixture of solvents for the anti-freezing purpose would be the mixture of glycerol and water. Glycerol is a non-toxic solvent commonly used in the food industry and pharmaceutical applications, and the mixture of glycerol of water can have a depressed freezing point of as low as −45°C at 66.7wt% glycerol/33.3wt% H_2_O (Lane, 1925) (Figure S11). We fabricated such a PVA gel (named aSC-GH) by soaking it in 66.7wt% glycerol/33.3wt% H_2_O after the same fabrication procedures as aSC gels. We conducted the tensile test to measure the mechanical properties of aSC-GH, which exhibits similar tensile strength but slightly lower modulus compared with aSC-DH. This mixture of glycerol and water also has anti-freezing ability, and when compared to the mixture of DMSO and water, it presents slightly lower performance in freezing point depression and mechanical strengthening (Figure S10). Nevertheless, it is still an acceptable alternative for fabricating an anti-freezing tough gel.

## STAR★Methods

### Key resources table

**Table:** 

REAGENT or RESOURCE	SOURCE	IDENTIFIER
**Chemicals, peptides, and recombinant proteins**		

Polyvinyl alcohol (Mw.89000-98000, 99% hydrolyzed)	Sigma Aldrich	Cat#341584; CAS: 9002-89-5
Mowiol 56-98 polyvinyl alcohol	Sigma Aldrich	Cat#10851; CAS: 9002-89-5
Sodium citrate dihydrate	Sigma Aldrich	Cat#W302600; CAS: 6132-04-3
Dimethyl sulfoxide	Sigma Aldrich	Cat#472301; CAS: 67-68-5
Sulfuric acid	Sigma Aldrich	Cat#258105; CAS: 7664-93-9
Glycerol	Fisher Scientific	Cat#G33-500; CAS: 56-81-5
Polyvinyl alcohol (Mw.88000-97000, 88% hydrolyzed)	Alfa Aesar	Cat#AA4123914; CAS: 9002-89-5
Sylgard 184	Dow	Cat#761036

### Resource availability

#### Lead contact

Further information and requests for resources should be directed to and will be fulfilled by the lead contact, Ximin He (ximinhe@ucla.edu).

#### Materials availability

The study did not generate any unique reagents.

### Method details

#### Materials

Polyvinyl alcohol (PVA) powder (Mw.89000-98000, 99% hydrolyzed), Mowiol 56-98 polyvinyl alcohol (PVA) powder (Mw.195000, 99% hydrolyzed), sodium citrate dihydrate, dimethyl sulfoxide (DMSO), and sulfuric acid were all purchased from Sigma Aldrich. Glycerol was purchased from Fisher Scientific. Polyvinyl alcohol (PVA) powder (Mw.88000-97000, 88% hydrolyzed) was purchased from Alfa Aesar. Sylgard 184 poly(dimethylsiloxane) (PDMS) was purchased from Dow. All chemicals were used without further purification.

#### Preparation of PVA precursors

PVA precursor solution was prepared by adding 10 wt% (15 wt% for the stiff part of the actuator) of PVA powder in water before heating up to 95°C and stirring for 2 hours.

#### Preparation of DMSO/H_2_O mixture

60 wt% of DMSO and 40 wt% of water were mixed to obtain DMSO/H_2_O mixture.

#### Preparation of sodium citrate solution

1.5 M sodium citrate solution was prepared by addition of 220 g of sodium citrate dihydrate in 500 mL of water, followed by stirring under 50°C for I hour.

#### Preparation of DMSO/H_2_SO_4_ mixture

DMSO/H_2_SO_4_ mixture was prepared by mixing 60 wt% of DMSO with 40 wt% of 2 M H_2_SO_4_ aqueous solution.

#### Preparation of glycerol/H_2_O mixture

Glycerol/H_2_O mixture was prepared by mixing 66.7 wt% of glycerol with 33.3 wt% of water.

#### Fabrication of PVA gels

Anisotropic sodium citrate>DMSO/H_2_O (aSC-DH) gel was fabricated by the following steps. 10 wt% PVA aqueous solution was first directionally frozen in an acrylic mold at -80°C for 3 hours. Then the piece of frozen material was soaked in a 1.5 M sodium citrate aqueous solution at room temperature for two days. After that, the gel was soaked in DMSO/H_2_O mixture at room temperature for 7 days. Three types of PVA powders (Mw.89000-98000, 99% hydrolyzed; Mw.195000, 99% hydrolyzed; Mw.88000-97000, 88% hydrolyzed) were used and their mechanical properties were compared. PVA with a higher molecular weight (Mw. 195000) yields stronger material, but because of the high viscosity of the precursor and the limited clamping force of our tensile test instrument, we choose to use PVA of lower molecular weight. PVA that is 88% hydrolyzed yields gels with much lower modulus and tensile strength, and forms gels in salt solution more slowly. Taking the convenience in fabrication and measurement, and the properties of the product into consideration, we used polyvinyl alcohol (PVA) powder (Mw.89000-98000, 99% hydrolyzed) to fabricate different types of PVA gels if not stated otherwise. Anisotropic freeze-thaw (a-freeze-thaw) gel was fabricated by directional freezing and thawing the 10 wt% PVA precursor for 5 cycles. Isotropic DMSO/H_2_O (iDH) gel was fabricated by mixing 10 wt% PVA in DMSO and 10 wt% PVA in water at a 3:2 ratio by weight. The mixture was then cooled in a -20°C freezer for 5 min. Anisotropic DMSO/H_2_O (aDH) gel was fabricated by the following steps. 10 wt% PVA aqueous solution was first directionally frozen in an acrylic mold at -80°C for 3 hours. Then the piece of frozen material was soaked in DMSO/H_2_O mixture precooled to -20°C for 2 days. Anisotropic sodium citrate (aSC) gel was fabricated by the following steps. 10 wt% PVA aqueous solution was first directionally frozen in an acrylic mold at -80°C for 3 hours. Then the piece of frozen material was soaked in a 1.5 M sodium citrate aqueous solution at room temperature for 2 days. Anisotropic sodium citrate>H_2_O (aSC-H) gel was fabricated by soaking aSC gel in water for two days. Anisotropic sodium citrate>DMSO/H_2_SO_4_ (aSC-DS) gel for conductivity measurements was fabricated by soaking the aSC gel in DMSO/H_2_SO_4_ mixture at room temperature for 7 days. Isotropic sodium citrate>DMSO/H_2_SO_4_ (iSC-DS) gel used as the control group in the demonstration of the anisotropic conductor was fabricated by the following steps. 10 wt% PVA precursors in water were first homogeneously frozen at -80°C. Then the gel was soaked in 1.5 M sodium citrate aqueous solution at room temperature for two days, and in DMSO/H_2_SO_4_ mixture at room temperature for 7 days. Anisotropic sodium citrate>DMSO/H_2_O>H_2_O (aSC-DH-H) gel for the control group in stiffness tests was fabricated by soaking the aSC-DH gel in water for 7 days. Isotropic sodium citrate>DMSO/H_2_O (iSC-DH-freeze-thaw) gel used as the control group in tear-resistance tests was fabricated by the following steps. 10 wt% PVA precursor in water first went through five freeze-thaw cycles. Then the gel was soaked in a 1.5 M sodium citrate aqueous solution at room temperature for two days, and in DMSO/H_2_O mixture at room temperature for 7 days. Isotropic sodium citrate>DMSO/H_2_O (iSC-DH-one-time-freezing) gel was fabricated by the following steps. 10 wt% PVA precursor in water was first homogeneously frozen at -80°C. Then the gel was soaked in a 1.5 M sodium citrate aqueous solution at room temperature for two days, and in DMSO/H_2_O mixture at room temperature for 7 days. Anisotropic sodium citrate>glycerol/H_2_O (aSC-GH) gel was fabricated by soaking the aSC gel in a glycerol/H_2_O mixture at room temperature for 7 days.

#### Fabrication of PVA actuator

Isotropic actuator: A two-piece mold was 3D printed with Creality Ender3. The PVA precursor was first poured into the mold, then it was frozen at -20°C and thawed at room temperature for 4 cycles with the lid on. The lid was then removed, and water was poured into the hollow chambers in PVA gel. After water was frozen at -20°C, a rod was inserted, and the precursor for the stiff PVA was poured into the mold. The whole thing then went through 2 freeze-thaw cycles before being taken out of the mold. In the final step, the actuator was soaked in a 1.5 M sodium citrate solution at room temperature for 2 days, then soaked in DMSO/H_2_O mixture at room temperature for 7 days.

Anisotropic actuator: A two-piece mold with no bottom was 3D printed with Creality Ender3, and a piece of glass slide was glued to the bottom of the mold. The PVA precursor was first poured into the mold, then it was directionally frozen in the vertical direction at -80°C with the lid on. The frozen precursor, together with the mold, were then soaked in a 1.5 M sodium citrate solution at room temperature for 1 day. After that, the bottom half of the actuator was taken out of the mold and placed top-down on a layer of stiff PVA precursor, which was then frozen at -20°C. Finally, the frozen gel was then soaked in DMSO/H_2_O mixture at room temperature for 7 days.

#### Fabrication of PDMS actuator

PDMS precursor was poured into the mold, then the mold was kept at 50°C with the lid on overnight. After the cured bottom half of the PDMS actuator was removed from the mold, the part was placed upside down on a petri dish filled with 3mm deep PDMS precursor. The top part was cured at 50°C overnight, and the actuator was cut out from the Petri dish-shaped PDMS block. Finally, holes were drilled with a long needle to create a channel that connects all hollow chambers.

#### Procedure for determining mechanical properties

Tensile tests were conducted using Cellscale Univert. All materials were cut into 2cm long (parallel or perpendicular to freezing direction), and 2mm wide bone-shaped samples (the thickness was determined by the fabrication steps). The strain rate was 1 mm/s. For low-temperature tests, a -45°C cooling chamber was placed around the sample, which kept the whole sample at -45°C during the elongation process. Young's modulus was obtained by calculating the initial slope of the stress-strain curve. Tensile strength was obtained by finding the highest point of the curve. Toughness was calculated by integrating the area under the curve. For tear-resistance tests, the isotropic and anisotropic PVA gels were cut halfway through in the middle before being stretched on Cellscale Univert. For the low-temperature stiffness tests, both aSC-DH and aSC-DH-H gels were cooled to -60°C. The samples were then placed across two Petri dishes, and a 50 g weight, also cooled to -60°C, was placed on top of the samples.

#### Procedure for observing the morphologies

aDH, aSC, and aSC-DH gels were soaked in DI water for two days before freeze-drying. The SEM images of dried samples were taken with ZEISS Supra 40VP SEM.

#### Procedure for measuring conductivities

Two samples were cut from the aSC-DS gel. Samples were immersed in a cooling bath in a water-proof plastic bag. AC impedance was measured with CHI660E Electrochemical Workstation at 10000HZ at various temperatures (20°C, 0°C, -20°C, -40°C, -60°C) in two directions (parallel and perpendicular). Conductivities were then calculated from the impedances.

#### Demonstration of anisotropic anti-freezing conductor

The anisotropic (in parallel direction) or the isotropic conductive gel was connected in a circuit with two antiparallel LEDs. Two temperature conditions are tested, namely room temperature and -20°C, where the -20°C condition was achieved by placing the conductive gels on a cooling plate. The applied voltage provided by CHI660E Electrochemical Workstation swept between -5V and 5V at 1V/s for 3 cycles. Videos were taken during the tests to compare the brightness of the LEDs at various voltages, and the C-V curves were measured using the same instrument.

#### Procedure for determining actuator performance

A 100 g weight was tied to the end of the actuators and a 30mL syringe was used to inject DMSO/H_2_O. In-air experiments were conducted both at room temperature and in a -45°C cooling chamber. Experiments in solution were conducted in DMSO/H_2_O at room temperature and DMSO/H_2_O precooled to -60°C.

#### Procedure for determining actuation force

A two-section PVA actuator was fabricated following the previously mentioned procedure. The actuator was placed under the loading cell of Cellscale Univert, with a petri dish attached to the loading cell, to collect the force generated by the actuator. The liquid was supplied through the syringe pump, and the pushing force created by the inflating actuator was recorded.

## Data Availability

No new code is generated and all original data are available from the authors upon request.

## References

[bib1] Cao J., Wang Y., He C., Kang Y., Zhou J. (2020). Ionically crosslinked chitosan/poly(acrylic acid) hydrogels with high strength, toughness and antifreezing capability. Carbohydr. Polym..

[bib2] Ahmed E.M. (2015). Hydrogel: preparation, characterization, and applications: a review. J. Adv. Res..

[bib3] Alsaid Y., Wu S., Wu D., Du Y., Shi L., Khodambashi R., Rico R., Hua M., Yan Y., Zhao Y., Aukes D., He X. (2021). Tunable sponge-like hierarchically porous hydrogels with simultaneously enhanced diffusivity and mechanical properties. Adv. Mater..

[bib4] Awan M., Buriak I., Fleck R., Fuller B., Goltsev A., Kerby J., Lowdell M., Mericka P., Petrenko A., Petrenko Y., Rogulska O., Stolzing A., Stacey G.N. (2020). Dimethyl sulfoxide: a central player since the dawn of cryobiology, is efficacy balanced by toxicity?. Regen. Med..

[bib5] Baldwin R.L. (1996). How Hofmeister ion interactions affect protein stability. Biophys. J..

[bib6] Benjamin M., Kaiser E., Milz S. (2008). Structure-function relationships in tendons: a review. J. Anat..

[bib7] Billiet T., Vandenhaute M., Schelfhout J., Van Vlierberghe S., Dubruel P. (2012). A review of trends and limitations in hydrogel-rapid prototyping for tissue engineering. Biomaterials.

[bib8] Catalán J., Díaz C., García-Blanco F. (2001). Characterization of binary solvent mixtures of DMSO with water and other cosolvents. J. Org. Chem..

[bib9] Cebon D., Cheung C.Y. (2015). Deformation mechanisms of bituminous materials. Adv. Asph. Mater. Road Pavement Constr..

[bib10] Chen C., Wang Y., Wu Q., Wan Z., Li D., Jin Y. (2020). Highly strong and flexible composite hydrogel reinforced by aligned wood cellulose skeleton via alkali treatment for muscle-like sensors. Chem. Eng. J..

[bib11] Cui Y., Gong H., Wang Y., Li D., Bai H. (2018). A thermally insulating textile inspired by polar bear hair. Adv. Mater..

[bib12] Hitchcock D.I., Dougan R.B. (1935). Freezing points of anti-coagulant salt solutions. J. Gen. Physiol..

[bib13] Hu X., Zhou J., Daniel W.F.M., Vatankhah-Varnoosfaderani M., Dobrynin A.V., Sheiko S.S. (2017). Dynamics of dual networks: strain rate and temperature effects in hydrogels with reversible H-bonds. Macromolecules.

[bib14] Hu O., Chen G., Gu J., Lu J., Zhang J., Zhang X., Hou L., Jiang X. (2020). A facile preparation method for anti-freezing, tough, transparent, conductive and thermoplastic poly(vinyl alcohol)/sodium alginate/glycerol organohydrogel electrolyte. Int. J. Biol. Macromol..

[bib15] Hua M., Wu S., Ma Y., Zhao Y., Chen Z., Frenkel I., Strzalka J., Zhou H., Zhu X., He X. (2021). Strong tough hydrogels via the synergy of freeze-casting and salting out. Nature.

[bib16] Ionov L. (2014). Hydrogel-based actuators: possibilities and limitations. Mater. Today.

[bib17] Lane L.B. (1925). Freezing points of glycerol and its aqueous solutions. Ind. Eng. Chem..

[bib18] Li J., Mooney D.J. (2016). Designing hydrogels for controlled drug delivery. Nat. Rev. Mater..

[bib19] Lin S., Liu J., Liu X., Zhao X. (2019). Muscle-like fatigue-resistant hydrogels by mechanical training. Proc. Natl. Acad. Sci. U S A.

[bib20] Liu Xinyue, Liu J., Lin S., Zhao X. (2020). Hydrogel machines. Mater. Today.

[bib21] Liu Xin, Zhang Q., Gao G. (2020). DNA-inspired anti-freezing wet-adhesion and tough hydrogel for sweaty skin sensor. Chem. Eng. J..

[bib22] Maganaris C.N., Narici M.V. (2005). Mechanical properties of tendons. Tendon Inj. Basic Sci. Clin. Med..

[bib23] Morelle X.P., Illeperuma W.R., Tian K., Bai R., Suo Z., Vlassak J.J. (2018). Highly stretchable and tough hydrogels below water freezing temperature. Adv. Mater..

[bib24] Owusu-Nkwantabisah S., Gillmor J., Bennett G., Slater G., Szakasits M., Rajeswaran M., Antalek B. (2018). Thermal stiffening of hydrophobic association hydrogels. Polymer (Guildf).

[bib25] Sadeghi R., Jahani F. (2012). Salting-in and salting-out of water-soluble polymers in aqueous salt solutions. J. Phys. Chem. B.

[bib26] Sato M., Hukuda S. (1962). Phase diagram for the system water–dimethylsulphoxide. J. Ceram. Assoc. Jpn..

[bib27] Scherzinger C., Schwarz A., Bardow A., Leonhard K., Richtering W. (2014). Cononsolvency of poly-N-isopropyl acrylamide (PNIPAM): microgels versus linear chains and macrogels. Curr. Opin. Colloid Interf. Sci..

[bib28] Steck J., Kim J., Yang J., Hassan S., Suo Z. (2020). Topological adhesion. I. Rapid and strong topohesives. Extrem. Mech. Lett..

[bib29] Sun J.-Y., Sun J.-Y., Zhao X., Zhao X., Illeperuma W.R.K., Illeperuma W.R.K., Chaudhuri O., Chaudhuri O., Oh K.H., Oh K.H., Mooney D.J., Mooney D.J., Vlassak J.J., Vlassak J.J., Suo Z., Suo Z. (2012). Highly stretchable and tough hydrogels. Nature.

[bib30] Tavakoli J., Tang Y. (2017). Hydrogel based sensors for biomedical applications: an updated review. Polymers (Basel).

[bib31] Theocaris P.S., Stassinakis C.A. (1981). Crack propagation in fibrous composite materials studied by SEM. J. Compos. Mater..

[bib32] Vila J., Franjo C., Pico J.M., Varela L.M., Cabeza O. (2007). Temperature behavior of the electrical conductivity of emim-based ionic liquids in liquid and solid states. Port. Electrochim. Acta.

[bib33] Wang W., Wang W., Liu Y., Liu Y., Wang S., Fu X., Fu X., Zhao T., Zhao T., Chen X., Chen X., Shao Z., Shao Z. (2020). Physically cross-linked silk fibroin-based tough hydrogel electrolyte with exceptional water retention and freezing tolerance. ACS Appl. Mater. Interfaces.

[bib34] Worland R., Block W., Rothery P. (1992). Survival of sub-zero temperatures by two South Georgian beetles (Coleoptera, Perimylopidae). Polar Biol..

[bib35] Wu S., Alsaid Y., Yao B., Yan Y., Zhao Y., Hua M., Wu D., Zhu X., He X. (2021). Rapid and scalable fabrication of ultra-stretchable, anti-freezing conductive gels by cononsolvency effect. EcoMat.

[bib36] Xu Y., Rong Q., Zhao T., Liu M. (2020). Anti-Freezing multiphase gel materials: bioinspired design strategies and applications. Giant.

[bib37] Yang C., Suo Z. (2018). Hydrogel ionotronics. Nat. Rev. Mater..

[bib38] Yang Y., Yang Y., Cao Y., Wang X., Chen Y., Liu H., Gao Y., Wang J., Liu C., Wang W., Yu J.K., Wu D. (2021). Anti-freezing, resilient and tough hydrogels for sensitive and large-range strain and pressure sensors. Chem. Eng. J..

[bib39] Yuk H., Lin S., Ma C., Takaffoli M., Fang N.X., Zhao X. (2017). Hydraulic hydrogel actuators and robots optically and sonically camouflaged in water. Nat. Commun..

[bib40] Zhang H., Hussain I., Brust M., Butler M.F., Rannard S.P., Cooper A.I. (2005). Aligned two- and three-dimensional structures by directional freezing of polymers and nanoparticles. Nat. Mater..

[bib41] Zhang S., Liu X., Barreto-Ortiz S.F., Yu Y., Ginn B.P., DeSantis N.A., Hutton D.L., Grayson W.L., Cui F.Z., Korgel B.A., Gerecht S., Mao H.Q. (2014). Creating polymer hydrogel microfibres with internal alignment via electrical and mechanical stretching. Biomaterials.

[bib42] Zuo Z., Zhang Y., Zhou L., Liu Z., Jiang Z., Liu Y., Tang L. (2021). Mechanical behaviors and probabilistic multiphase network model of polyvinyl alcohol hydrogel after being immersed in sodium hydroxide solution. RSC Adv..

