# Supplementary material for: Tendon-inspired anti-freezing tough gels
Source: iScience. 2021 Aug 17;24(9):102989. doi: 10.1016/j.isci.2021.102989 (PMC8417335; doi:10.1016/j.isci.2021.102989)
Supplement: Document S1. Figures S1–S12 [file mmc1.pdf]

iScience, Volume *24*

Supplemental information

Tendon-inspired anti-freezing tough gels

Sidi Duan, Shuwang Wu, Mutian Hua, Dong Wu, Yichen Yan, Xinyuan Zhu, and Ximin He

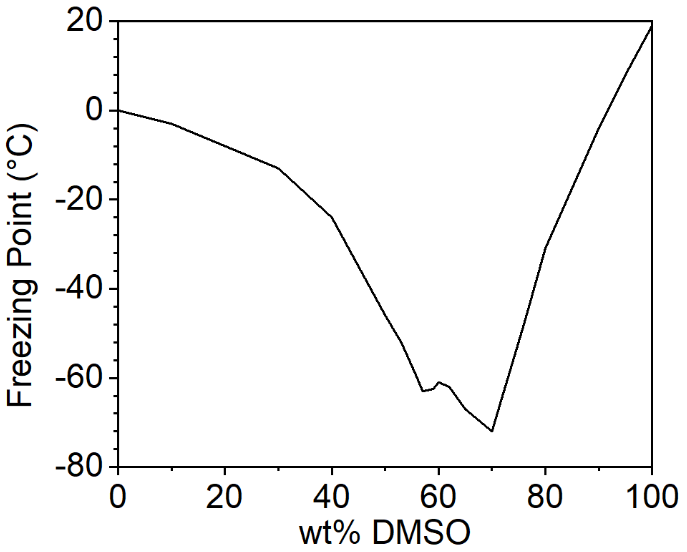

Figure S1. Phase diagram of DMSO-water mixture (SATO and HUKUDA, 1962). Related to STAR Methods.

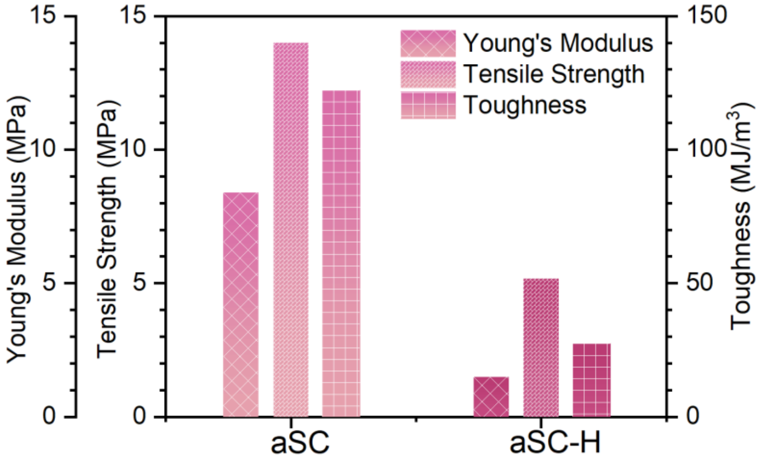

Figure S2. Young’s modulus, tensile strength, and toughness of aSC and aSC-H gels. Related to Figure 2

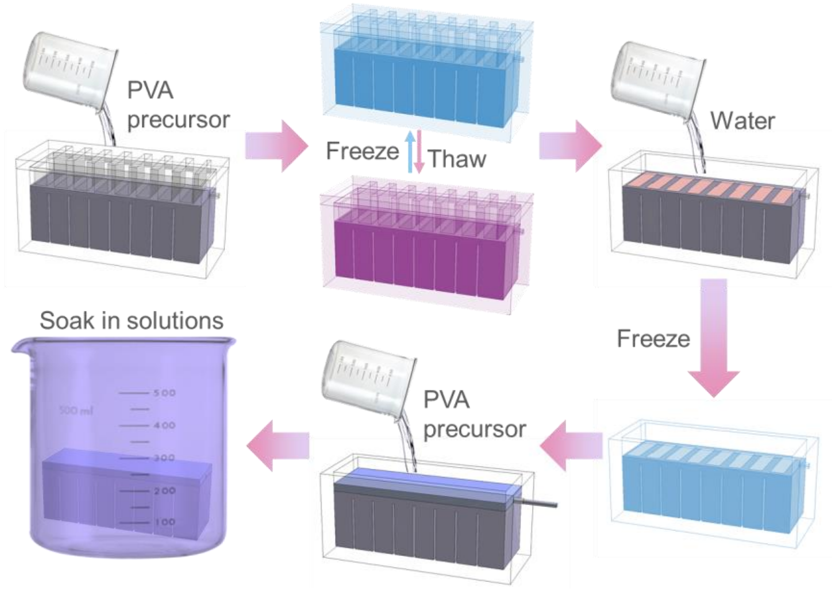

Figure S3. Fabrication procedure of PVA hydraulic actuator. Related to STAR Methods.

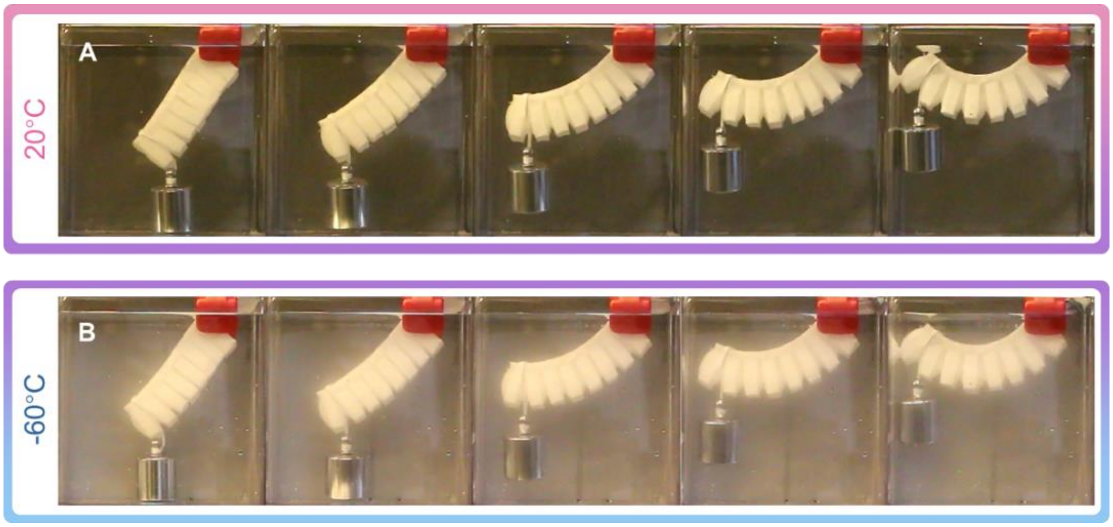

Figure S4. Photos of PVA actuator lifting 100 g weight in DMSO/H_2O mixture at 20°C and -60°C. Related to Figure 4.

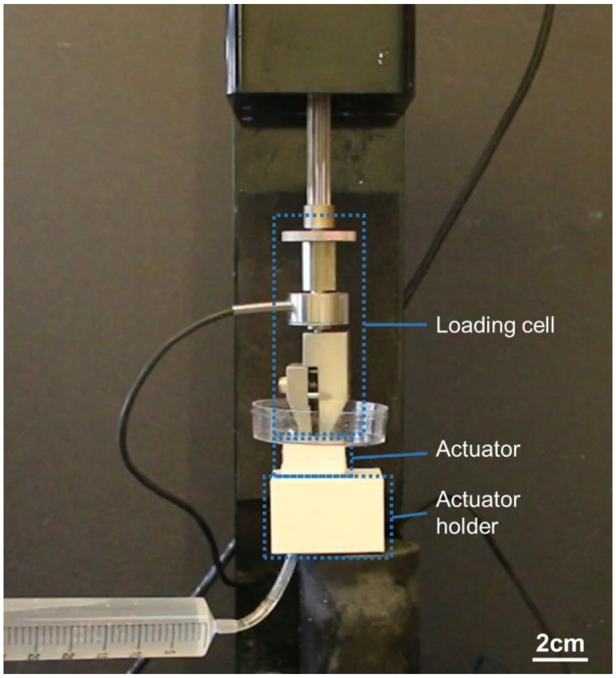

Figure S5. Setup for actuation force measurement. Related to STAR Methods.

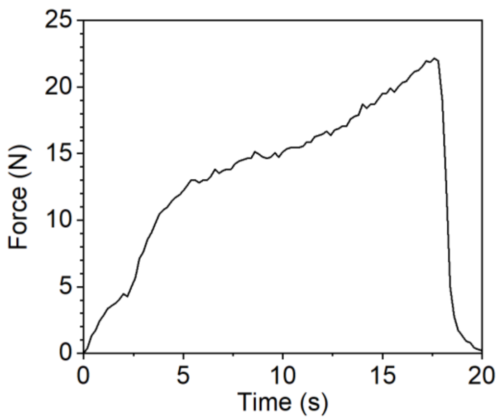

Figure S6. Test result for actuation for measurement. The two-section actuator failed at 22N. Related to Figure 4.

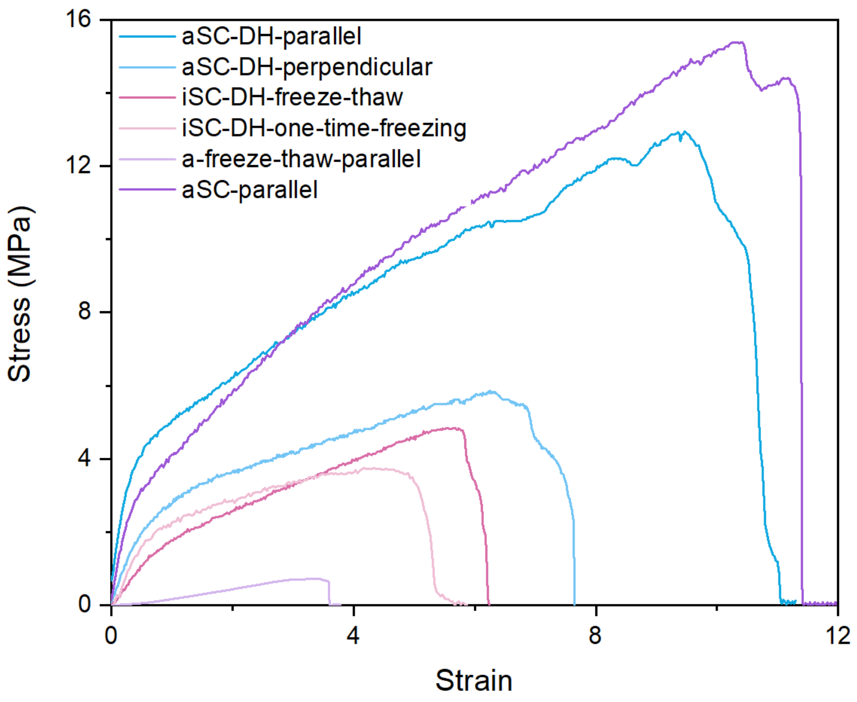

Figure S7. Tensile test results for aSC gel in the parallel direction, aSC-DH gel in the parallel and perpendicular directions, iSC-DH gel with 5 homogeneous freeze-thaw cycles (iSC-DH-freeze-thaw), iSC/DH gel with one-time homogeneous freezing (iSC-DH-one-time-freezing), and PVA gel that only went through 5 directional freezing-thawing cycles (a-freeze-thaw-parallel). Related to Figure 2.

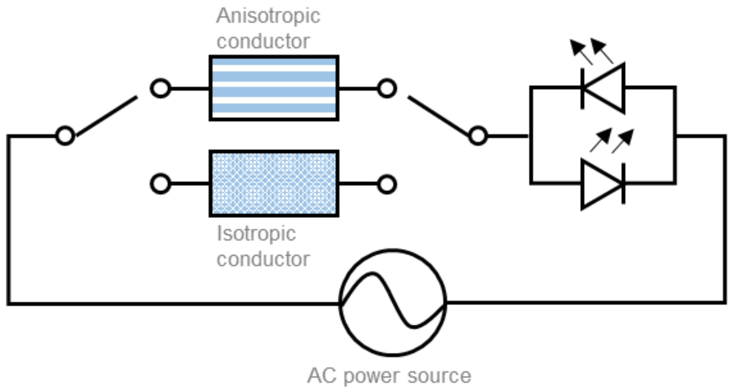

Figure S8. Illustration for the circuit containing LEDs, anisotropic conductor, and isotropic conductor. Related to Figure 3.

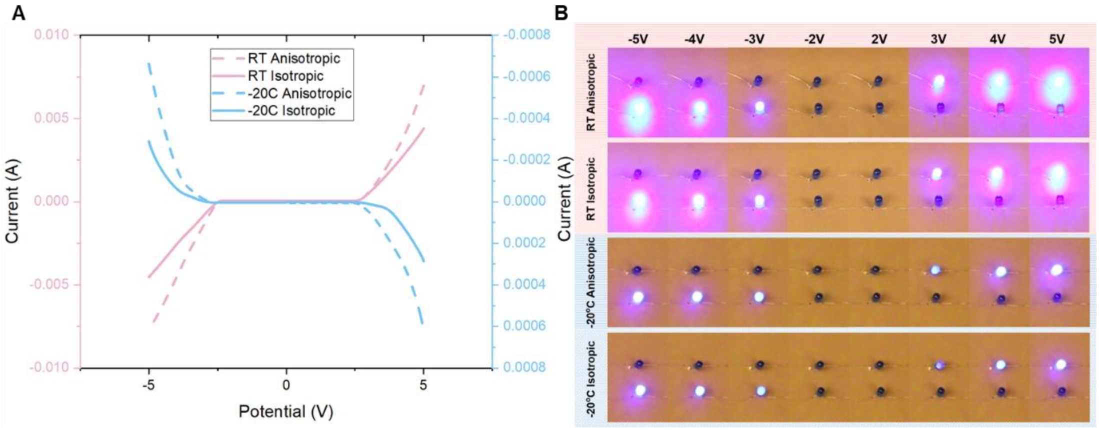

Figure S9. (A) C-V curves for the circuits with anisotropic or isotropic gels as conductors operating at RT or -20°C. (B) Photos of LED lights at various applied voltages. Related to Figure 3.

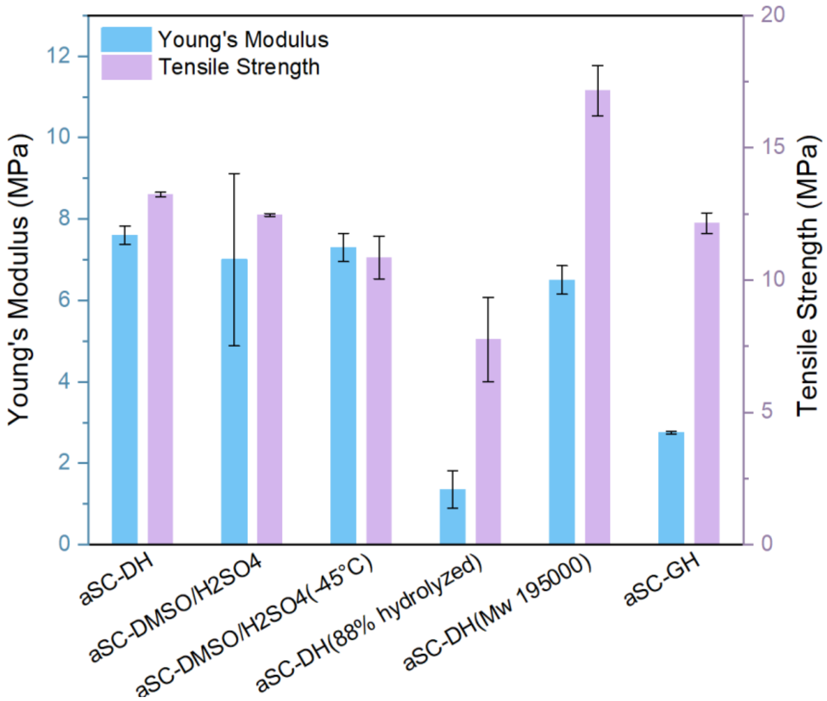

Figure S10. Young's moduli and tensile strengths of aSC-DH, aSC-DMSO/H_2SO_4 (at room temperature and -45°C), aSC-DH (88% hydrolyzed), aSC-DH (Mw 195000), and aSC-GH gels. Related to Figure 3 and STAR Methods.

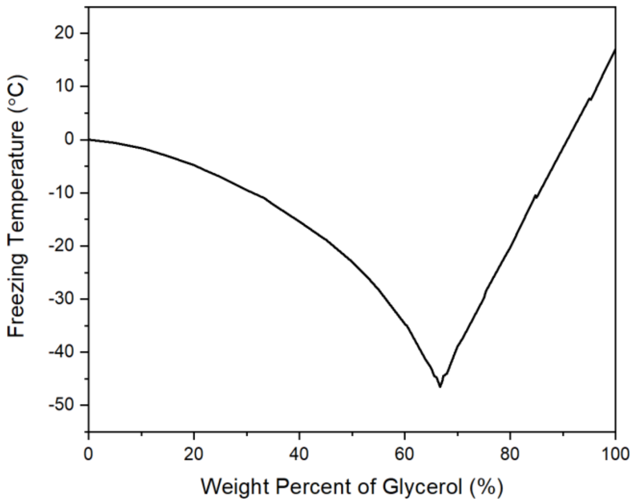

Figure S11. Freezing point vs glycerol content for the mixture of glycerol and water (Lane, 1925). Related to STAR Methods.

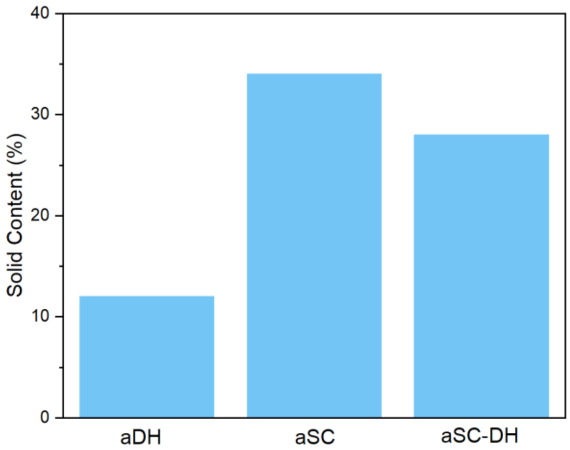

Figure S12. The solid contents of aDH, aSC, and aSC-DH gels. Related to Figure 2.
